# MicroRNA-143 targets DNA methyltransferases 3A in colorectal cancer

**DOI:** 10.1038/sj.bjc.6605195

**Published:** 2009-07-28

**Authors:** E K O Ng, W P Tsang, S S M Ng, H C Jin, J Yu, J J Li, C Röcken, M P A Ebert, T T Kwok, J J Y Sung

**Affiliations:** 1 Department of Medicine and Therapeutics, Institute of Digestive Disease, Li Ka Shing Institute of Health Sciences, The Chinese University of Hong Kong, Shatin, Hong Kong SAR, China; 2 Department of Biochemistry, The Chinese University of Hong Kong, Shatin, Hong Kong SAR, China; 3 Department of Surgery, The Chinese University of Hong Kong, Hong Kong SAR, China; 4 Institute of Pathology, Department of Pathology, Charite University Hospital, Berlin, Germany; 5 Department of Medicine II, Technical University of Munich, Munich, Germany

**Keywords:** miR-143, DNMT3A, colorectal cancer, tumour suppressor

## Abstract

**Background:**

MicroRNAs (miRNAs) are 19-25-nucleotides regulatory non-protein-coding RNA molecules that regulate the expressions of a wide variety of genes, including some involved in cancer development. In this study, we investigated the possible role of miR-143 in colorectal cancer (CRC).

**Methods:**

Expression levels of human mature miRNAs were examined using real-time PCR-based expression arrays on paired colorectal carcinomas and adjacent non-cancerous colonic tissues. The downregulation of miR-143 was further evaluated in colon cancer cell lines and in paired CRC and adjacent non-cancerous colonic tissues by qRT–PCR. Potential targets of miR-143 were defined. The functional effect of miR-143 and its targets was investigated in human colon cancer cell lines to confirm miRNA–target association.

**Results:**

Both real-time PCR-based expression arrays and qRT–PCR showed that miR-143 was frequently downregulated in 87.5% (35 of 40) of colorectal carcinoma tissues compared with their adjacent non-cancerous colonic tissues. Using *in silico* predictions, DNA methyltranferase 3A (DNMT3A) was defined as a potential target of miR-143. Restoration of the miR-143 expression in colon cell lines decreased tumour cell growth and soft-agar colony formation, and downregulated the DNMT3A expression in both mRNA and protein levels. DNMT3A was shown to be a direct target of miR-143 by luciferase reporter assay. Furthermore, the miR-143 expression was observed to be inversely correlated with DNMT3A mRNA and protein expression in CRC tissues.

**Conclusion:**

Our findings suggest that miR-143 regulates DNMT3A in CRC. These findings elucidated a tumour-suppressive role of miR-143 in the epigenetic aberration of CRC, providing a potential development of miRNA-based targeted approaches for CRC therapy.

Colorectal cancer (CRC) is the third most common cancer worldwide, with an estimated 1 million new cases and half a million deaths each year (Parkin *et al*, 2005). Screening for CRC allows the early diagnosis of the malignancy and reduces the mortality of the disease (Walsh and Terdiman, 2003). With the advent of new chemotherapeutic agents, such as angiogenesis inhibitor and TGF-*α* inhibitors, there is growing interest to identify new prognostic biomarkers and therapeutic targets for this disease.

MicroRNAs are 19-25-nucleotides regulatory non-protein-coding RNA molecules that regulate the expressions of a wide variety of genes by sequence-specific base pairing on the 3′-untranslated regions (3′UTRs) of target mRNA, resulting in mRNA degradation or inhibition of translation. Patterns of miRNA expression are meticulously regulated and have important roles in oncogenesis (He *et al*, 2005; Lu *et al*, 2005; Calin and Croce, 2006). Over the last decade, the number of human genes that are known to be regulated by miRNAs has increased rapidly (He and Hannon, 2004; Chen, 2005). Studies have shown that profiles of miRNA expression differ between normal and tumour tissues, which vary among tumour types (He *et al*, 2005; Lu *et al*, 2005; Calin and Croce, 2006). The downregulation of miRNA subsets implies a tumour-suppressor function, which is often observed in tumour development; for example, downregulated let-7 in lung cancer (Takamizawa *et al*, 2004; Johnson *et al*, 2005; Yanaihara *et al*, 2006) suppresses *Ras* (Johnson *et al*, 2005), deleted or downregulated miR-15 and miR-16 in chronic lymphocytic leukemia suppress *BCL2* (Calin *et al*, 2002; Cimmino *et al*, 2005), and miR-17-5p and miR-20a control the balance of cell death and proliferation driven by the proto-oncogene *c-Myc* (O'Donnell *et al*, 2005). At present, more than 690 human miRNAs are annotated in the miRBase registry (miRBase version 12.0), but most of the genes regulated by human miRNAs are not well defined.

Studies have examined miRNA expression profiles of CRC compared with those of normal colonic mucosa. Two independent studies reported a general downregulation of miRNAs in tumour cells in which the expression levels of two miRNAs, miR-145 and miR-143, were significantly reduced in colorectal tumour cells (Calin *et al*, 2004; Gregory and Shiekhattar, 2005). Bandrés *et al* (2006) also evaluated the expression of 156 miRNAs by real-time PCR in colon cancer cell lines, as well as in paired CRC tissues, and found that the deregulation of some identified miRNAs in CRC tumours was consistent with previous studies. In this study, we aimed at investigating the role of a downregulated miRNA, miR-143, in CRC.

## Materials and methods

### Cell culture

Seven human colon cancer cell lines, including 228, CaCO2, Clone A, HCT116, HT-29, MIP101, and SW480 (American Type Culture Collection, Manassas, VA, USA), were cultured at 37 °C in a 10% CO_2_ atmosphere and maintained routinely in Dulbecco's modified Eagle's Medium (DMEM) supplemented with 10% fetal bovine serum and 2 mM L-glutamine (Invitrogen, Carlsbad, CA, USA).

### Patient samples

A total of 50 pairs of primary CRCs and their paired non-cancerous colonic tissues (obtained from 30 patients from Hong Kong and 20 patients from Germany) were collected. All samples were collected from patients who underwent a surgical resection of tumours, either at the Prince of Wales Hospital (Hong Kong) or at the Charité University Hospital (Germany). All patients provided written informed consent for the use of their tissues. This project was approved by the Joint CUHK-NTE Clinical Research Ethics Committee (Hong Kong) and Ethics Committee at the Charité University Hospital (Germany).

All tissues had been histologically confirmed to be an adenocarcinoma of the colon. Tissue samples were collected, snap-frozen in liquid nitrogen, and stored at −80 °C until further analysis. For real-time PCR-based miRNA profiling analysis, 10 pairs of CRC and adjacent non-cancerous colonic tissues obtained from Hong Kong patients were used. The remaining 40 paired tissue samples were used for quantitative PCR (qPCR) validation.

### Real-time PCR-based miRNA array

The miRNA expression profiling was performed using cancer microRNA qPCR Array with the QuantiMir system (System Biosciences, Mountain View, CA, USA). This system is a real-time PCR-based array containing a panel of 95 cancer-related and well-established mature miRNA assays and the *U6* transcript as a normalisation signal. The kit contains all reagents and primers for polyadenylation, reverse transcription, and qPCR, except for reagents for qPCR. In brief, 1 *μ*g of total RNA containing small RNA extracted from tissue samples was first polyadenylated by poly(A) polymerase and then reverse transcribed to cDNA using a mixture of oligo-dT adaptor provided in the kit. The cDNA then serves as the template for SYBR real-time PCR using Power SYBR Master Mix (Applied Biosystems, Foster City, CA, USA), using miRNA-specific primers provided by the manufacturer. SYBR PCR was performed in an ABI PRISM 7500 Fast Real-time PCR system (Applied Biosystems). ΔCt was calculated by subtracting the Ct values of U6 from the Ct values of the gene of interest. ΔΔCt was then calculated by subtracting ΔCt of the control from ΔCt of the sample. Fold change of the gene was calculated by the equation: 2^−ΔΔCt^.

### Real-time qPCR

Total RNA containing small RNA was extracted from tissues and cell lines by TRIzol reagent (Invitrogen), according to the manufacturer's instructions. For miRNA qPCR, reverse transcription was performed using the QuantMir RT Kit (System Biosciences). In brief, 1 *μ*g of RNA containing miRNA was polyadenylated by poly(A) polymerase and then reverse transcribed to cDNA using oligo-dT primers. The cDNA then serves as the template for SYBR real-time PCR using SYBR-green PCR Master Mix (Applied Biosystems). The miR-143-specific forward primer sequence was 5′-TGAGATGAAGCACTGTAGCTC-3′ and was designed on the basis of miRNA sequences obtained from the miRBase database. Human U6 snRNA was used for normalisation. For DNA methyltranferase 3A (*DNMT3A*) mRNA qPCR, cDNA was synthesised with oligo-dT primers and MMLV reverse transcriptase (Promega Corporation, Madison, WI, USA), according to the manufacturer's instructions. Gene-specific primers for the DNMT3A gene were adapted from Xiong *et al* (2005). mRNA expression was normalised to *β*-actin. All real-time qPCR assays were carried out by the PRISM 7500 Fast Real-time PCR system (Applied Biosystems). The amplification profile was denatured at 95 °C for 10 min, followed by 40 cycles of 95 °C for 15 s and 60 °C for 1 min, in which fluorescence was acquired. At the end of the PCR cycles, melting curve analyses were performed, as well as electrophoresis of the products on 3.5% agarose gels to validate the specific generation of the expected PCR product. ΔCt was calculated by subtracting the Ct values of U6 or *β*-actin from the Ct values of the gene of interest. ΔΔCt was then calculated by subtracting ΔCt of the control from ΔCt of the sample. Fold change of gene was calculated using the Equation 2^−ΔΔCt^.

### Ectopic miR-143 expression

An enforced expression of the miR-143 expression in colon cancer cells was achieved by transfection with miR-143 precursor (Ambion, Austin, TX, USA). Cells were plated in culture dishes or in 24 of 96-well plates for 24 h and transfected with 40 nM precursor with Lipofectamine 2000 (Invitrogen) for 24 h. Commercially available precursor control (Ambion) was used as a negative control. Cells were then subjected to further assays or to RNA/protein extraction.

### Cell proliferation assay

Cell proliferation was measured by 3-[4,5-dimethylthiazol-2-yl]-2, 5-diphenyltetrazolium bromide (MTT) assay. Cells were seeded in a 96-well plate for 24 h, transfected with miR-143 precursor or DNMT3A siRNA for 24 h, and further cultured in normal medium for 5 days. Thereafter, the cells were incubated in 0.1 mg ml^−1^ MTT at 37 °C for 3 h and lysed in DMSO (dimethyl sulfoxide) at room temperature for 30 min. The absorbance in each well was measured at 580 nm by a microplate reader.

### Anchorage-independent colony formation assay

Soft-agar plates were prepared in 24-well plates with a bottom layer of 0.6% Noble agar in serum-free DMEM. Cells were trypsinised, and 500 cells were seeded onto the bottom layer after being mixed with 0.3% Noble agar in DMEM supplemented with 10% fetal calf serum. Plates were incubated in a 37-°C incubator for 3 weeks. The number of colonies was counted after staining with 0.05% crystal violet for 1 h and washed extensively with 1 × PBS.

### DNMT3A silencing by siRNA

The sense sequence of a siRNA oligonucleotide targeting *DNMT3A* transcript was used as follows: 5′-CAGGAGAUGAUGUCCAACCC-3′ (Ambion). Scrambled siRNA was used as a negative control. Cells were plated in culture dishes or in 96-well plates for 24 h, and transfected with 40 nM siRNA and Lipofectamine 2000 (Invitrogen) for 24 h. The cells were then subjected to further assays or to RNA extraction.

### Western blot analysis

Cells were lysed in Lammeli's lysis buffer, resolved in SDS–PAGE minigel, and transferred onto an Immobilon-P membrane (Millipore, Billerica, MA, USA). The membranes were probed with 1 : 1000 diluted primary antibodies against DNMT3A (Epigentek, Brooklyn, NY, USA) at room temperature for 2 h, washed extensively with 0.1% Tween-20 in PBS, and incubated with secondary antibodies conjugated with horseradish peroxidase (1 : 10 000 dilution). Signals were visualised with enhanced chemiluminescence (Amersham Life Science Inc., Little Chalfont, UK).

### Luciferase activity assay

The 3′UTR of DNMT3A containing an intact miR-143 recognition sequence was amplified and the PCR product was sub-cloned into a pGL3 vector (Promega Corporation) immediately downstream of the luciferase gene. A pGL3 construct containing DNMT3A 3′UTR with point mutations in seed sequence was synthesised using a site-directed mutagenesis kit (Stratagene, La Jolla, CA, USA), according to the manufacturer's instructions. Cells were co-transfected with 800 ng pGL3 constructs with or without miR-143 precursor for 24 h. Each sample was co-transfected with 0.05 *μ*g pRL-CMV plasmid expressing Renilla luciferase to monitor transfection efficiency (Promega Corporation). Luciferase activity assay was performed 24 h after transfection using the dual-luciferase reporter assay system (Promega Corporation). Relative luciferase activity was normalised with Renilla luciferase activity.

### Statistical analysis

Expressions of miR-143 in paired colorectal tumours and adjacent non-cancerous tissues were compared by paired *t-*test. The difference between the two groups in the MTT assay, anchorage-independent soft-agar assay, and luciferase reportor assay was analysed by two-sided Student's *t-*test. Data are expressed as the mean±s.d. from at least three independent experiments. All *P*-values are two-sided and a value of <0.05 was considered to be statistically significant. All statistical calculations were performed using SPSS software (version 13.0, SPSS, Chicago, IL, USA).

## Results

### Validation of downregulated miR-143 in CRC tissues and human colon cancer cell lines

**Figure 1:**
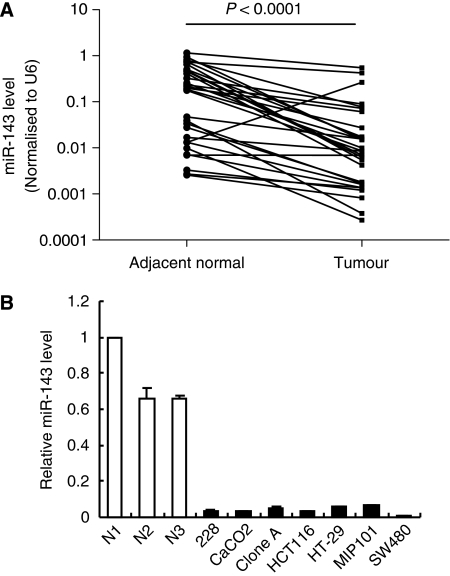
The downregulated miR-143 expression in both primary colorectal cancer tissues and colon cancer cell lines. (**A**) The relative miR-143 expression between tumour tissues and their paired adjacent colonic normal tissues from 30 CRC patients using real-time qPCR. Expression of miR-143 (Log_10_ scale at Y axis) was normalised to U6. Statistical difference was analysed using Wilcoxon's test, *P*<0.0001. (**B**) The relative miR-143 expression in colon cancer cell lines was much lower than that of the three non-cancerous colonic tissues (N1, N2, and N3).

To confirm miR-143 downregulation in CRC, we quantified miR-143 levels by both real-time PCR-based array and qRT–PCR. Using real-time PCR-based miRNA expression profiling arrays containing a panel of 95 cancer-related and well-established mature miRNAs (including miR-143), we compared the miRNA profile of pooled RNA samples of primary CRC tumour tissues from 10 Hong Kong Chinese patients with pooled RNA samples of their paired adjacent colonic non-cancerous tissues. With a cutoff value of a three-fold difference, 33 of 95 (35%) miRNAs were downregulated in CRC. Of the 33 downregulated miRNAs, the 10 most downregulated miRNAs, including miR-125a, miR-125b, miR-133a, miR-137, miR-143, miR-145, miR-204, miR-215, miR-26a, and miR-30a-5p, are shown in Table 1. On the basis of the profiling results, miR-143 is the sixth most downregulated miRNA, with a seven-fold downregulation. We further validated the miR-143 expression level in primary CRC tumour tissues from 20 German and 10 Hong Kong patients by qRT–PCR. Our results showed that miR-143 level was significantly decreased in 27 of 30 (90%) CRC tumour tissues when compared with that in their adjacent normal tissues (fold change ranging from −3 to −120, *P*<0.0001; Wilcoxon's paired test; Figure 1A). Of the 20 CRC patients from Germany, 19 (95%) had a miR-143 downregulation. Of the 10 CRC patients from Hong Kong, 8 (80%) had a miR-143 downregulation. The expression of miR-143 was further determined in a panel of seven human colon cancer cell lines (HT-29, SW480, 228, CaCO2, Clone A, HCT116, and MCP101). The expression of miR-143 was markedly lower in all of the seven colon cancer cell lines than in the three non-tumour colonic tissues (Figure 1B).

### *In silico* prediction of miR-143 target

**Figure 2:**
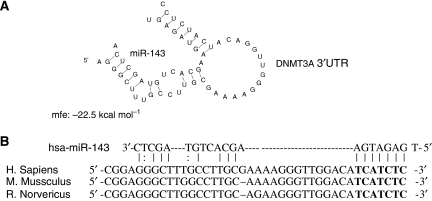
*In silico* prediction of the miR-143 target. (**A**) Predicted binding of miR-143 (grey) to DNMT3A 3′UTR (black). (**B**) Sequence alignment of human miR-143 with different species of DNMT3A 3′UTR. The seed sequence of miR-143 (bold) perfectly matches with the 3′UTR of DNMT3A.

Using the algorithms for target gene prediction, including PicTar (Lall *et al*, 2006), TargetScan (Lewis *et al*, 2003), and miRanda (John *et al*, 2004), the key enzyme in DNA methylation, DNMT3A, was identified as one of the potential targets of miR-143. The predicted binding of miR-143 with *DNMT3A* 3′UTR is illustrated in Figure 2A. The sequence alignment of human miR-143 with different species of *DNMT3A* 3′UTR was also conserved (Figure 2B), indicating that *DNMT3A* is one of the potential direct targets of miR-143.

### Ectopic miR-143 expression inhibits CRC cell growth and downregulates DNMT3A

**Figure 3:**
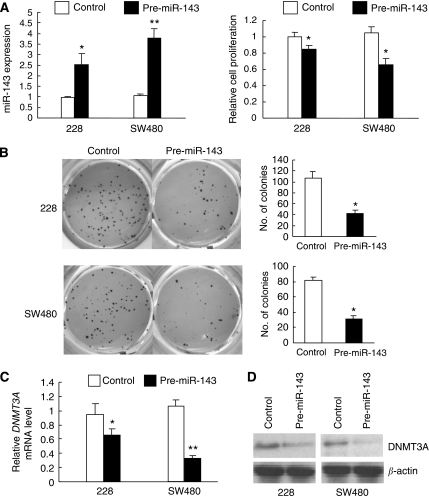
The functional effect of ectopic miR-143 expression in 228 and SW480 cells. (**A**) Ectopic miR-143 expression suppresses cell proliferation in colon cancer cells. Cells were transfected with miR-143 precursor or control precursor for 24 h. Cell proliferation at 5 days after transfection was assessed by MTT assay. The miR-143 expression was examined by qPCR (Mann–Whitney test, ^*^P<0.05, ^**^P<0.01). Relative cell proliferation was compared with the corresponding control inhibitor or precursor (Mann–Whitney test, ^*^P<0.05). (**B**) Ectopic miR-143 expression reduced the anchorage-independent growth of cancer cells using soft-agar colony formation assay. Cells were plated in 0.3% noble agar for 3 weeks. The number of colonies was counted after staining with 0.05% crystal violet (Mann–Whitney test, ^*^P<0.05). Ectopic miR-143 expression reduced both (**C**) mRNA and (**D**) protein expression of DNMT3A. Cells were transfected with miR-143 precursor or with control precursor for 24 h, and then lysed for RNA or protein extraction. *DNMT3A* mRNA was detected by real-time qPCR (Mann–Whitney test, ^*^P<0.05, ^**^P<0.01) and protein expression was detected by western blotting with an anti-DNMT3A antibody. *β*-Actin was used as a loading control.

Frequent downregulation of miR-143 in colon cancer cell lines and primary CRC carcinomas implies that miR-143 may have a role in CRC carcinogenesis. To prove this, the effect of ectopic expression of miR-143 on cell growth was investigated in two colon cancer cell lines (228 and SW480). The rationale of using these two cell lines is that both cell lines expressed a relatively low miR-143 level among the seven cell lines (Figure 1B). As shown in Figure 3A, the increased expression of miR-143 by ectopic miR-143 expression significantly inhibited the growth of 228 and SW480 cells (all *P*-values <0.05; Mann–Whitney test). Furthermore, miR-143 expression affected not only tumour cell growth but also malignant transformation phenotypes, as featured by the anchorage-independent growth of cancer cells in soft-agar medium. The enforced miR-143 expression significantly reduced the clone formation efficiency of 228 and SW480 cells in soft agar (all *P*-values <0.05; Mann–Whitney test; Figure 3B). These results provided strong evidence that miR-143 has a role in suppressing tumour cell growth. Our data indicated that an enforced miR-143 expression led to a dramatic reduction of DNMT3A expression at both the mRNA (*P*<0.05 for 228 cells and *P*<0.01 for SW480 cells; Mann–Whitney test; Figure 3C) and protein levels (Figure 3D), suggesting a potential regulation of DNMT3A by miR-143.

### DNMT3A is the direct target miR-143

**Figure 4:**
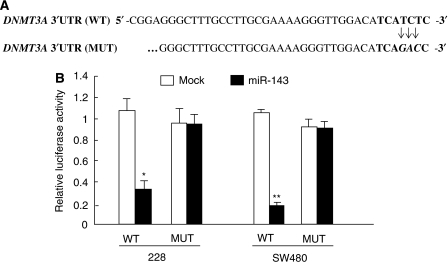
DNMT3A is the direct target of miRNA-143. (**A**) The wild-type (WT) and mutated (MUT) 3′UTR of DNMT3A, with the seed region (bold) and base substitutions (bold and italic), were sub-cloned into luciferase reporter construct and are shown. (**B**) Ectopic miR-143 expression inhibits wild-type but not mutant DNMT3A 3′UTR reporter activity in 228 and SW480 cells. Cells were co-transfected with miR-143 precursor and with either WT or MUT DNMT3A 3′UTR reporter construct. Luciferase activity assay was performed at 24 h after transfection (Mann–Whitney test, ^*^P<0.05, ^**^P<0.01).

To further confirm that DNMT3A is the direct target of miR-143, a segment of the 3′UTR of *DNMT3A*, with or without point mutations in the seed sequence (Figure 4A), was sub-cloned downstream of the firefly luciferase reporter. The constructs were then co-transfected with miR-143 precursor or with pre-miR control for luciferase activity assays. The relative luciferase activity of the WT construct of *DNMT3A* 3′UTR in both the colon cancer cells was significantly reduced in the presence of miR-143 (*P*<0.05 for 228 cells and *P*<0.01 for SW480 cells; Mann–Whitney test), whereas such a suppressive effect of miR-143 on luciferase activity was not observed in both cells with the MUT construct of DNMT3A 3′UTR (Figure 4B), highlighting a direct and specific interaction of miR-143 on *DNMT3A* 3′UTR.

### Knockdown expression of DNMT3A reduced colon cancer cell growth

**Figure 5:**
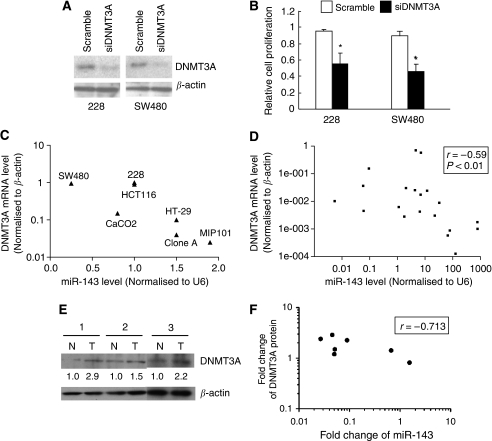
Relationship between miR-143 and DNMT3A. Knockdown of DNMT3A inhibited cell proliferation in 228 and SW480 cells. Cells were transfected with DNMT3A siRNA for 24 h. Transfection with scramble siRNA was used as negative control. (**A**) The DNMT3A protein level 5 days after transfection was examined by western blot. The experiment has been repeated thrice. (**B**) Cell proliferation 5 days after transfection was assessed using MTT assay. Relative cell proliferation was compared with the corresponding scramble siRNA transfection by the Mann–Whitney test, ^*^P<0.01. (**C**) The scatter plot of expression correlation between miR-143 and *DNMT3A* mRNA in seven colon cancer cell lines. Inverse correlation was obtained using Spearman's correlation, *r*=−0.78, *P*<0.05. (**D**) The scatter plot of expression correlation between miR-143 and *DNMT3A* mRNA in 10 paired adjacent normal and CRC tissues. Inverse correlation was also obtained by Spearman's correlation, *r*=−0.59, *P*<0.01. (**E**) The DNMT3A protein level was semi-quantified using western blot analysis of CRC tumours (T) and of adjacent normal cells (N). (**F**) The scatter plot of the fold changes of the miR-143 and DNMT3A protein (Log_10_ scale at both X axis and Y axis) in seven paired CRC samples (Spearman's correlation, *r*=0.71, *P*<0.05).

To further establish a link between miR-143 and its downstream target, DNMT3A, tumour cell proliferation after a siRNA-mediated knockdown of DNMT3A was examined. Consistent with the growth inhibitory effect of miRNA-143, cell proliferation of 228 and SW480 cells was significantly reduced by 42 and 44%, respectively, once the DNMT3A protein expression was effectively suppressed by siRNA (all *P*-values <0.05; Mann–Whitney test; Figure 5A and B).

### Expression relationship between miR-143 and DNMT3A in primary CRC tissues

To confirm the relevance of the expression of DNMT3A and the relationship between miR-143 and DNMT3A, we assessed the expressions of miR-143 and *DNMT3A* mRNA in the seven colon cancer cell lines and in an independent set of human CRC tumour tissues and their adjacent colonic tissues from 10 CRC Hong Kong patients. As shown in Figure 5C, an inverse correlation of expression between miR-143 and *DNMT3A* mRNA was observed in all colon cancer cell lines (*r*=−0.78, *P*=0.048; Spearman's correlation). Of the CRC patients who were analysed, 80% (8 of 10) simultaneously showed a downregulation of miR-143 and an upregulation of DNMT3A in tumour tissues compared with their paired non-cancerous colonic tissues. We showed that expressions between miR-143 and DNMT3A mRNA were inversely correlated in all 10 paired CRC and adjacent normal tissues (*r*=−0.59, *P*=0.0066; Spearman's correlation; Figure 5D). More importantly, we also found an inverse correlation of DNMT3A protein and miR-143 in seven paired CRC tissues (*r*=−0.71, *P*<0.05; Spearman's correlation; Figure 5E and F).

## Discussion

Studies have reported that miR-143 downregulation was common in various cancers (Michael *et al*, 2003; Akao *et al*, 2006, 2007; Slaby *et al*, 2007). In this study, we confirmed that miR-143 was frequently downregulated in CRC tissues than in their corresponding non-cancerous colonic tissues, as illustrated in Figure 1A. Importantly, we showed for the first time that miR-143 exerted its function by specifically targeting the gene of a key enzyme, DNMT3A, involved in DNA methylation. Furthermore, we showed that miR-143 expression was inversely correlated with DNMT3A expression in CRC. In addition to a previous study that reported that the miR-29 family regulated both DNMT3A and DNMT3B in lung cancer (Fabbri *et al*, 2007), in this study we showed that DNMT3A is regulated by miR-143 in CRC. This suggested that target gene may be regulated by different miRNAs in different tumours.

Our results obtained from profiling arrays and qPCR validation were in concordance with previous studies that showed that miR-143 was downregulated in both colon cancer cell lines and in more than 85% of CRC patients from Hong Kong (*n*=30) and Germany (*n*=20). These additional data provide evidence that miR-143 downregulation in CRC commonly occurred in different ethnic groups. Of the CRC tissues analysed by qPCR analysis, our data indicated that fold changes of miR-143 expression between CRC tumours and the corresponding adjacent normal tissues were not associated with patient characteristics, such as gender (*P*=0.563, *χ*^2^) and tumour stage (*P*=0.718, *χ*^2^) (data not shown). Accordingly, the frequent downregulation of miR-143 in CRC prompted us to believe that miR-143 may have a tumour-suppressive role in CRC development. By restoring miR-143 expression in colon cancer cells, we indeed showed that miR-143 suppressed both cell growth and soft-agar malignant transformation in colon cancer cells, suggesting a tumour-suppressive role of miR-143.

Our findings indicated that there is a vital molecular link between miR-143 and DNMT3A. First, we showed that restoration of miR-143 expression downregulated DNMT3A expression in both mRNA and protein levels. Second, both loss-of-function study of DNMT3A by siRNA-mediated knockdown and gain-of-function study of miR-143 by enforced miR-143 expression produced a suppressive effect on tumour cell growth, suggesting that their effects on cellular transformation are inversely correlated. Third, the inverse correlation between miR-143 and DNMT3A expression in both colon cancer cell lines and human CRC tissues further consolidates that downregulation of miR-143 resulting in an upregulation of DNMT3A is significant in CRC development. More importantly, we also provide evidence from the luciferase activity assay that DNMT3A is a direct target of miR-143. Taken together, our findings confirmed that miR-143 regulates DNMT3A expression and has a tumour-suppressive role in CRC development.

It is well known that DNA methylation has an important role in oncogenesis. One of the common features in carcinogenesis is the silencing of tumour suppressor genes by hypermethylation. Specific changes in DNA methylation patterns in human cancers could be useful in specific targets for treatment (Jones and Baylin, 2007). Methylation changes to the genome are controlled by DNA methyltransferases (DNMTs). At present, three catalytically active DNMTs, namely DNMT1, DNMT3A, and DNMT3B, have been identified (Jeltsch, 2002). All DNMTs possess *de novo* methylation activity, but DNMT1 is inefficient in *de novo* methylation. During DNA replication, the DNA methylation pattern is maintained by DNMT1 after the methylation pattern has been established (Pradhan *et al*, 1999). Emerging studies found that the levels of *DNMT1*, *DNMT3A*, and *DNMT3B* mRNA are reportedly increased in various malignancies, including colorectal, liver, and gastric cancers (Eads *et al*, 1999; Oh *et al*, 2007; Ding *et al*, 2008). Previous studies have shown that gastrointestinal cancer was characterised by high levels of DNMTs and a low demethyltransferase expression (Fang *et al*, 2006). DNMTs and demethyltransferase cooperated with each other, and led to genetic instability (Geiman *et al*, 2004) that eventually promoted cancer progression (Fang *et al*, 2004). More recently, a study showed that a high level of DNMT3A protein expression was significantly associated with a lower overall survival in lung cancer (Fabbri *et al*, 2007). Thus, patients with a higher DNMT3A expression had shorter overall survival. Although DNMT1 and DMNT3B are also important in cancer development, we did not test the effects of miR-143 on DMNT1 and DMNT3B, because our *in silico* predictions did not highlight DNMT1 or DMNT3B as the potential binding target of miR-143.

Although a high number of oncogenes were predicted to be the putative target of miR-143 shown in internet algorithms, it has only been reported that miR-143 regulated the extracellular signal-regulated kinase 5 (ERK5) expression (Esau *et al*, 2004; Akao *et al*, 2006), and downregulation of miR-143 in cancer cells may be directly involved in carcinogenesis through the activation of the mitogen-activated protein kinase cascade through ERK5. On the other hand, the reactivation of tumour suppressor genes by demethylation could represent another mechanism through which miR-143 exerts its tumour suppressor function by repressing DNMT3A. Thus, further study should address the relative contribution of these two mechanisms to promote anti-oncogenic effect.

Several issues should be addressed in the future. First, as the number of samples for studies on miR-143 downregulation and DMNT3A–miR-143 expression correlation is still small, further validations in large cohorts and in independent studies are necessary. Second, owing to the heterogeneity of CRC (e.g., high-level DNA microsatellite instability (MSI-H) *vs* MSI-low or CpG island methylator phenotype-high (CIMP-high) *vs* CIMP-low), it would be interesting to examine which subsets of CRCs are more dependent on miR-143 downregulation. Moreover, there may be a link between CIMP-high and DNMT3A. Third, additional studies will need to investigate the regulatory mechanism of miR-143 expression so as to better understand why miR-143 is frequently downregulated in CRC.

In conclusion, miR-143 was frequently downregulated in CRC and is a potential tumour suppressor miRNA for CRC development. miR-143 regulates DNMT3A and might have a part in the regulation of DNA methylation. These findings may provide a potential development of miRNA-based targeted approaches for the treatment of CRC.

## Figures and Tables

**Table 1:** Ten most downregulated miRNAs in colorectal cancer

**MicroRNA**	**MirBase No.**	**Fold changes**
hsa-miR-30a-5p	MIMAT0000087	−26.3
hsa-miR-145	MIMAT0000437	−25.5
hsa-miR-137	MIMAT0000429	−20.6
hsa-miR-133a	MIMAT0000427	−10.8
hsa-miR-204	MIMAT0000264	−8.8
hsa-miR-143	MIMAT0000435	−7.0
hsa-miR-215	MIMAT0000271	−6.7
hsa-miR-26a	MIMAT0000082	−6.3
hsa-miR-125b	MIMAT0000423	−6.1
hsa-miR-125a	MIMAT0000443	−6.0
